# The Angiotensin Type 1 Receptor Antagonist Losartan Prevents Ovariectomy-Induced Cognitive Dysfunction and Anxiety-Like Behavior in Long Evans Rats

**DOI:** 10.1007/s10571-019-00744-x

**Published:** 2019-10-21

**Authors:** Glenda V. Campos, Aline M. A. de Souza, Hong Ji, Crystal A. West, Xie Wu, Dexter L. Lee, Brittany L. Aguilar, Patrick A. Forcelli, Rodrigo C. de Menezes, Kathryn Sandberg

**Affiliations:** 1 Department of Medicine, Georgetown University, Suite 232 Building D, 4000 Reservoir Road, NW, Washington, DC 20057 USA; 2 Department of Pharmacology and Physiology, Georgetown University, Washington, DC USA; 3 Department of Physiology, Howard University, Washington, DC USA; 4 Department of Biological Sciences, Federal University of Ouro Preto, Ouro Preto, Brazil

**Keywords:** Cognition, Memory, Postmenopausal, Premature ovarian failure

## Abstract

Women who have bilateral oophorectomies prior to the age of natural menopause are at increased risk of developing mild cognitive decline, dementia, anxiety, and depressive type disorders. Clinical and animal studies indicate angiotensin type 1 receptor (AT_1_R) blockers (ARBs) have blood pressure (BP)-independent neuroprotective effects. To investigate the potential use of ARBs in normotensive women at increased risk of developing neurocognitive problems, we studied a rat model of bilateral oophorectomy. Long Evans rats were sham-operated (Sham) or ovariectomized (Ovx) at 3 months of age and immediately treated continuously with vehicle (Veh) or the ARB losartan (Los) for the duration of the experiment. In contrast to many hypertensive rat models, ovariectomy did not increase mean arterial pressure (MAP) in these normotensive rats. Ovariectomized rats spent less time in the open arms of the elevated plus maze (EPM) [(% total time): Veh, 34.1 ± 5.1 vs. Ovx, 18.7 ± 4.4; *p* < 0.05] and in the center of the open field (OF) [(s): Veh, 11.1 ± 1.7 vs. Ovx, 6.64 ± 1.1; *p* < 0.05]. They also had worse performance in the novel object recognition (NOR) test as evidenced by a reduction in the recognition index [Veh, 0.62 ± 0.04 vs. Ovx, 0.45 ± 0.03; *p* < 0.05]. These adverse effects of ovariectomy were prevented by Los. Losartan also reduced plasma corticosterone in Ovx rats compared to Veh treatment [(ng/mL): Ovx–Veh, 238 ± 20 vs. Ovx–Los, 119 ± 42; *p* < 0.05]. Ovariectomy increased AT_1_R mRNA expression in the CA3 region of the hippocampus (Hc) [(copies x 10^6^/µg RNA): Sham–Veh, 7.15 ± 0.87 vs. Ovx–Veh, 9.86 ± 1.7; *p* < 0.05]. These findings suggest the neuroprotective effects of this ARB in normotensive Ovx rats involve reduction of plasma corticosterone and blockade of increased AT_1_R activity in the hippocampus. These data suggest ARBs have therapeutic potential for normotensive women at increased risk of developing cognitive and behavioral dysfunction due to bilateral oophorectomy prior to the natural age of menopause.

## Introduction

The Mayo Clinic Cohort Study of Oophorectomy and Aging showed that the risk of mild cognitive impairment, dementia, anxiety, and depression nearly doubled in women who underwent bilateral oophorectomy prior to the age of natural menopause, regardless of the indication for oophorectomy (e.g., benign ovarian condition or for cancer prophylaxis) (Rocca et al. 2007, 2008). These first large-scale studies of cognition and mood disorders with long-term follow-up also showed that the risk of these neurocognitive disorders increased the younger the age at oophorectomy. These findings from women living in the United States were confirmed in a Danish historical cohort study, published in 2010, that queried national disease registries (Phung et al. 2010). The Danish study showed that the risk of dementia with onset before the age of 50, increased in women who underwent bilateral oophorectomy prior to the age of natural menopause, again, with the risk increasing the younger the age at surgery. In 2014, a study of two large and well-characterized cohorts in the United States further documented the positive association between bilateral oophorectomy prior to the age of natural menopause and cognitive decline including Alzheimer’s disease (AD) neuropathology (Rocca et al. 2016). In addition, the Religious Orders Study and Rush Memory and Aging Project found a faster rate of global cognitive decline, especially episodic memory and semantic memory, the earlier the age of surgical menopause whereas no associations were observed in women who had natural menopause (Bove et al. 2014).

Bilateral oophorectomy before the onset of natural menopause causes an abrupt cessation of estrogen production with a consequent drop in circulating levels of 17β-estradiol (E_2_). Controversy, however, over the harm versus benefit of estrogen replacement therapy (ERT) for neurologic diseases continues (O’Hagan et al. 2012). The Women’s Health Initiative showed that women who initiated ERT alone or in combination with a progestin in the late postmenopausal stage (ages 65–79 years) experienced an increased risk of dementia and cognitive decline regardless of the presence or absence of progestin (Shumaker et al. 2004). Three observational studies, however, suggest that the neuroprotective versus harmful effects of estrogen depend on the age at time of treatment initiation and on the stage of menopause (Rocca et al. 2010). Furthermore, a randomized, double-blind, placebo-controlled trial in healthy women showed that ERT for 5 years did not benefit or harm verbal episodic memory, executive function, or global cognition regardless of time since menopause (Henderson et al. 2016). In contrast, the Mayo Clinic Cohort Study of Oophorectomy reported a failure of ERT to prevent the risk of anxiety and depressive outcomes (Rocca et al. 2008). Even if ERT is proven to provide neurocognitive benefit, it is contraindicated in some women (e.g., because of active thrombosis, endometriosis, or history of breast cancer) and others may not elect ERT for other reasons. Thus, it is imperative to develop preventive therapeutic strategies for women at increased risk of neurocognitive disorders due to premenopausal bilateral oophorectomy.

Several clinical studies have shown angiotensin receptor blockers (ARBs) have neuroprotective effects. Patients within the UK general practice database with antihypertensive prescription data and who were diagnosed with dementia (1214) or AD (5797) had fewer prescriptions for ARBs compared to other antihypertensive medications (Davies et al. 2011). In a Department of Veterans Affairs population of over 370,000 individuals who had diabetes, the risk of developing dementia was reduced in those treated with ARBs compared to other antihypertensive therapy (Johnson et al. 2012). In another large (> 810,000) US Veteran cohort, the hazard rates for incident dementia was smallest in the ARB treatment group compared to other antihypertensives and in patients with pre-existing AD, there was a lower risk of admission to a nursing home in the ARB treatment group compared to other antihypertensive medications (Li et al. 2010).

Studies in animals support these clinical findings. ARBs were shown to protect cognition (Pelisch et al. 2011) and reduce anxiety- and depressive-like behaviors (Saavedra et al. 2006) from hypertension and stroke (Faure et al. 2006), traumatic brain injury (Timaru-Kast et al. 2012) and isolation stress (Saavedra et al. 2006). However, little is known regarding the potential of ARBs to exert neurocognitive protection in women who had bilateral oophorectomies prior to the natural age of menopause or in animal models of sudden ovarian hormone loss. In this study, we investigated the effect of ovariectomy on cognitive function and anxiety- and depressive-like behavior in Long Evans rats as well as the ability of the ARB losartan (Los) to prevent neurocognitive impairments induced by ovariectomy.

## Methods

### Animals

Female Long Evans rats were purchased from Harlan/Envigo (Indianapolis, USA) at 3 months of age. They were housed in pairs in a temperature and humidity-controlled room under a light/dark cycle of 12 h. Food and water were provided ad libitum. Body weight, food, and water intake were measured weekly.

### Surgery

Sham and ovariectomy surgeries were conducted in 3 month old rats. The surgery was performed under 2.5% isoflurane anesthesia (Patterson Veterinary, Greeley, CO) at 1 L/min oxygen (Roberts Oxygen Company Inc, Rockville, MD). Bilateral flank incisions were made. In the Ovx group, the ovaries were removed, while in the Sham group, the ovaries were exposed without excising them. Then, the muscle layers and skin were individually sutured. All rats received the analgesic carprofen (5 mg/kg (Rimadyl); Zoetis; Parsippany, NJ) subcutaneously after surgery and up to 72 h when needed.

### Drug Treatment

Immediately after surgery, the Sham and Ovx rats were randomized and half were given vehicle (drinking water; filtered tap water) while the other half were treated with Los (Sigma, St. Louis, MO) dissolved in the drinking water. The dose of losartan (10 mg/kg/day) remained constant until the day of euthanasia by adjusting the concentration of drug in the drinking water based on daily water consumption and BW, as we previously described (Ji et al. 2007).

### Experimental Design

**Fig. 1:**
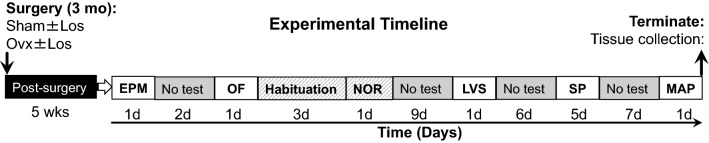
Experimental timeline of behavioral testing. At 3 months of age, animals were sham-operated (Sham) or ovariectomized (Ovx) and immediately treated continuously with vehicle (Veh) or losartan (Los) throughout the duration of the experimental protocol. Five weeks after the surgeries were completed, behavioral testing was initiated. Thereafter, mean arterial pressure (MAP) was measured and tissues were collected and used for further analyses. *mo* months, *EPM* elevated plus maze, *OF* open field, *NOR* novel object recognition, *LVS* looming visual stimuli, *SP* sucrose preference

Five weeks after surgery and in the presence of continuous drug treatment, the animals were exposed to a battery of behavioral tests with at least a two-day interval between each test, as illustrated in Fig. 1. In order to habituate and reduce the stress response due to manipulation of the experimenter, the animals were gently handled for 5 min a day for one week prior to the first behavioral test. Animals were transported from the animal facility to the testing room at least 30 min before initiating a test. All apparatuses were cleaned with a 70% ethanol solution between each behavioral test session. After all testing was complete, the rats were anesthetized with isoflurane and MAP was measured. Then, the animals were euthanized for plasma and tissue collection.

### Elevated Plus Maze

The EPM test was conducted as previously described (Pellow et al. 1985). The apparatus consists of two opposed open arms and two perpendicular arms enclosed by walls (50 cm × 10 cm × 40 cm; Bioseb In Vivo Research Instruments, Pinellas Park, FL) raised 50 cm above the floor. The test was performed under 115 lx illumination and was scored by EthoVision XT software (Noldus Information Technology, Leesburg, VA). The distance traveled during the testing was used as an assessment of spontaneous locomotor activity. Anxiety-like behavior was assessed by measuring the number of entries and percentage of time spent exploring the open arms over a 5-min period.

### Open Field

The OF test was conducted as previously described (Prut and Belzung 2003). The apparatus consists of a square arena (90 cm × 90 cm × 40 cm; Bioseb In Vivo Research Instruments). The test was performed under 115 lx illumination and was scored by EthoVision XT software (Noldus Information Technology). The distance traveled during the testing was used as an assessment of spontaneous locomotor activity. Anxiety-like behavior was determined by measuring the number of entries and amount of time spent exploring the center portion of the field (45 cm × 45 cm) over a 5-min period.

### Novel Object Recognition

The NOR test was adapted from Drumond et al. and Zanini et al. (Drumond et al. 2012; Zanini et al. 2017). This test uses the same apparatus and software (EthoVision XT; Noldus Information Technology) as for the OF test. Rats spent 10 min/day for 3 days exploring the apparatus without any stimulus in order to become habituated. On the test day, rats were placed in the center of the arena and allowed to explore two identical objects for 10 min (training phase). After 60 min, one of the objects was replaced by a new one and the animals were allowed to explore for another 5 min (test phase). The time spent exploring the objects in the training and test phases were scored, and the recognition index was obtained by the following equation: time exploring the new object/total exploration time of the two objects in the test phase. Values above 0.5 indicate preference for the new object. Exploratory behavior was defined as the amount of time spent smelling and touching the object and when the animal’s nose approached the object within 2 cm.

### Looming Visual Stimuli

The LVS test was conducted as previously described (Aguilar et al. 2018). The test uses a transparent cylindrical chamber (43.5 cm × 18.5 cm) with a computer screen located above the chamber (Dell, Round Rock, TX). All testing was conducted under 20 lx red light. Initially, the computer screen was completely gray for a 2 min baseline period. During the stimulus period, the screen displayed a black dot that rapidly expanded from 2° to 20° of visual angle over 250 ms. After reaching maximum size, the dot remained stable for 250 ms before disappearing. The expanding dot was presented consecutively 15 times over 22 s, mimicking the visual stimulus of a predator attack (Wei et al. 2015). After stimulus presentation, the gray screen was again presented until the experiment ended at 3 min (poststimulus period). Videos were truncated into equivalent length periods (22 s each) and manually scored for freezing behavior by a blinded observer using ANYMaze software (Stoelting Co., Wood Dale, IL). Freezing was defined as ceasing all activity, maintaining an attentive attitude with head raised, eyes open, and body remaining in the same position.

### Sucrose Preference

The SP test was conducted as previously described (Aguilar et al. 2018; Prut and Belzung 2003). The preference for sweet solutions over tap water was assessed by measuring the intake of sucrose solution (1% sucrose—w/v) and tap water over 5 days as follows: 4 consecutive days of a 2-h exposure period to the choice of sugar water vs plain. On the last day of the test, rats were water restricted for 2 h followed by a 2-h exposure period to the preference test. During the test phase, animals were housed in individual cages with water and sucrose solution bottles (200 mL each) placed in random positions. Once the test phase was completed, the animals were returned to their original cages. Sucrose preference was based on the following equation: total sucrose consumption (mL)/[total sucrose consumption (mL) + total water consumption (mL)].

### Mean Arterial Pressure

Terminal BP measurements were determined as we previously described (Zheng et al. 2008). Briefly, animals were anesthetized with 2.5% isoflurane at 1 L/min oxygen. A polyethylene catheter was inserted into the femoral artery and arterial pressures were collected every minute for 40 min using a BP Analyzer (Digi-Med, Louisville, KY).

### Plasma and Tissue Collection

After BP measurements were completed, each animal was euthanized and then blood was collected by cardiac puncture most often in the morning. Plasma was isolated from blood by centrifugation and stored at − 80 °C until further use. The uterus was collected and weighed and the brain was rapidly removed and placed in dry ice for fast cooling and then stored at − 80 °C until further processing. Subsequently, 300-μm-thick coronal brain sections were obtained by using a cryostat (Leica CM 1850, Leica Biosystems Inc., Buffalo Grove, IL). The sections were placed on histological slides kept in contact with dry ice. Biopsy punches of the basolateral amygdala (BLA) and CA1 and CA3 regions of the hippocampus (Hc) were collected and stored at -80 °C until mRNA was isolated from these tissues.

### Angiotensin Type 1 Receptor mRNA Expression

AT_1_R mRNA was measured by real-time PCR. Total RNA from the CA1 & CA3 regions of the Hc and the BLA were extracted using an RNAqueous™-Micro Total RNA Isolation Kit (Thermo Fisher Scientific, Waltham, MA). First-strand cDNA was made from 500 ng of total RNA using a iScript cDNA synthesis kit (BioRad, Hercules, CA) with Moloney Murine Leukemia Virus (MMLV) RNase H + reverse transcriptase, oligo(dT) and random hexamers. RNA concentrations were measured on a NanoDrop 2000 (Thermo Scientific). Quantitation of specific mRNAs was performed by real-time PCR using the ABI StepOnePlus real-time PCR (Applied Biosystems Inc., Foster City, CA). The real-time PCR mixture consisted of RNase-free water, TaqMan Fast Advance Master Mix (Thermo Fisher Scientific), and the following specific primers (300 nM) and probe (10 µM): forward primer: rAT_1_R-CR-330F 5′-CAACCTCTACGCCAGTGTGTTC-3′; reverse primer: rAT_1_R-CR-470R:5′-CCAGCCATCAGCCAGATGA-3′; and probe: rAT_1a_R-CR-382T: Fam-CTGGCCATCGTCCACCCAATGAAGT-Tamra and cDNA samples. PCRs without reverse transcription and no template were included to control for genomic DNA contamination. The standard curve with rAT_1a_R plasmid DNA was used to determine the DNA copy number in the Hc and BLA samples. The data were expressed in DNA copy numbers × 10^6^ and normalized to µg of total RNA in each sample.

### Plasma Corticosterone

Corticosterone was determined in the plasma using the corticosterone ELISA Kit (Neogen, Lexington, KY). Briefly, plasma samples were diluted and added to the microplate and incubated with an enzyme conjugate for 1 hour at room temperature. The plate was then washed to remove unbound material. The bound enzyme conjugate was detected by the addition of substrate, which generated an optimal color after 30 min. Quantitative test results were obtained by measuring and comparing the absorbance reading of the sample wells to the standards using a FLUOstar Omega plate reader (BMG LABTECH Inc., Cary, NC) at 650 nm.

### Statistical Analysis

Statistical comparisons were assessed using GraphPad Prism software (version 8, GraphPad Inc., La Jolla, CA). The effect of ovariectomy was analyzed by Student’s *t* test in both the Veh and Los treatment groups. Two-way ANOVA was used to analyze the effects of ovariectomy and Los treatment. In the NOR and LVS tests, each phase was analyzed individually. Data were expressed as mean ± standard error of the means. Significance was defined as *p* < 0.05.

## Results

### Effect of Ovariectomy and Losartan on Uterine Wet Weight and Body Weight

The rodent uterotrophic bioassay is a well-validated test for E_2_ deficiency (Kleinstreuer et al. 2016). Thus, to confirm that the bilateral ovariectomies were successful, we measured the uterine wet weight (WW) at the end of the behavioral testing. Ten weeks after ovariectomy, uterine WWs decreased by 80% regardless of treatment (*p* < 0.0001) (Table 1). Initial BWs did not differ across all four groups; however, ovariectomy increased BW gain in both the Veh- and Los-treated groups ten weeks after ovariectomy (*p* < 0.0001) (Table 1). Losartan had no effect on uterine WW or BW in either the Sham or Ovx animals.

**Table 1:** Effect of ovariectomy and Los on uterine WW, BW, MAP, and plasma corticosterone

Animal group	Uterine WW (g)	Initial BW (g)	Final BW (g)	BW gain (final/initial)	MAP (mm Hg)	Plasma corticosterone (ng/mL)
Sham–Veh	0.15 ± 0.01	259 ± 5.1	290 ± 6.6	1.12 ± 0.01	103 ± 4.2	180 ± 32
Ovx–Veh	0.03 ± 0.002*	265 ± 4.8	338 ± 7.6*	1.28 ± 0.02*	98.1 ± 2.0	238 ± 20
Sham–Los	0.14 ± 0.008	252 ± 2.6	280 ± 3.3	1.11 ± 0.01	90.2 ± 1.7^#^	186 ± 45
Ovx–Los	0.03 ± 0.001*	264 ± 1.9	328 ± 4.3*	1.24 ± 0.01*	88.3 ± 1.1^#^	119 ± 42*

Rats were sham-operated (Sham) or ovariectomized (Ovx) at 3 months of age and treated with vehicle (Veh) or losartan (Los) for 10 weeks before the animals were euthanized and uterine WW, BW, MAP, and plasma corticosterone were determined. For uterine WW and BW: Sham–Veh (*n* = 14), Ovx–Veh (*n* = 12), Sham–Los (*n* = 14), and Ovx–Los (*n* = 12). For MAP: Sham–Veh (*n* = 6), Ovx–Veh (*n* = 6), Sham–Los (*n* = 12), and Ovx–Los (*n* = 12). For plasma corticosterone: Sham–Veh (*n* = 10), Ovx–Veh (*n* = 10), Sham–Los (*n* = 10), and Ovx–Los (*n* = 10). *p < 0.05 vs Sham, same treatment (Student’s *t* test); ^#^*p* < 0.05 vs. vehicle, same surgery (Student’s *t* test). Regardless of treatment, ovariectomy decreased WW (*p* < 0.0001 by two-way ANOVA (surgery, treatment) F = 248; DFn = 1; DFd = 48) and increased BW gain (*p* < 0.0001 by two-way ANOVA (surgery, treatment), F = 131; DFn = 1; DFd = 56). Losartan reduced the MAP in both Sham and Ovx rats (*p* < 0.0001 by two-way ANOVA (surgery, treatment) F = 30.1; DFn = 1; DFd = 32)

### Effect of Ovariectomy and Losartan on Mean Arterial Pressure

MAP measured by indwelling catheter in anesthetized Long Evans rats did not increase ten weeks after ovariectomy (Table 1). The animals remained normotensive. Thus, we were able to use this rat strain to study the effects of ovariectomy on behavior independently of BP. Losartan treatment reduced the MAP in both Sham and Ovx rats (*p* < 0.0001) (Table 1); however, this reduction in MAP did not cause hypotension (≈ MAP < 80 mm Hg) and basal MAP was considered within normal limits.

### Effect of Ovariectomy and Losartan on Elevated Plus Maze Behavior

**Fig. 2:**
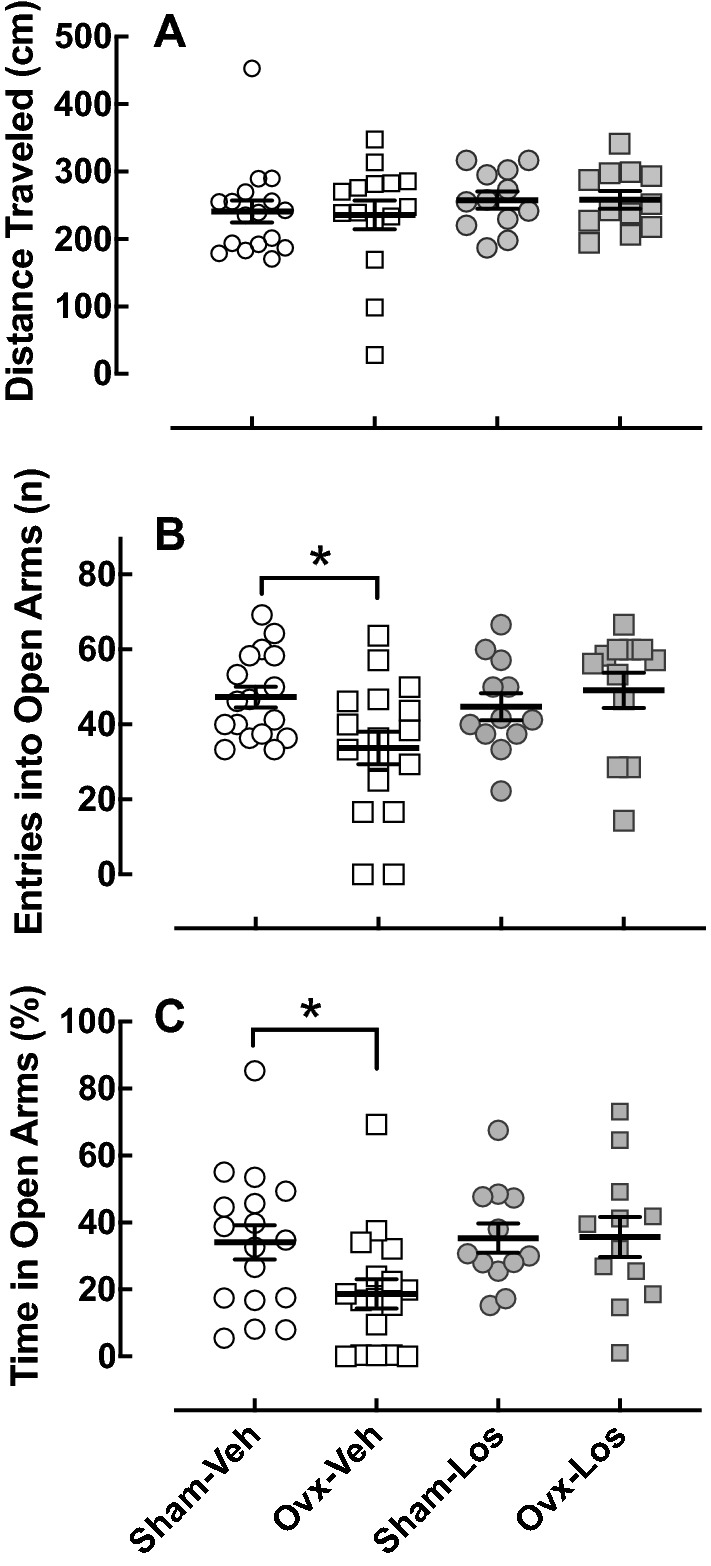
Effect of ovariectomy and losartan on behavior in the elevated plus maze. EPM was assessed in Sham–Veh (*n* = 17), Ovx–Veh (*n* = 17), Sham–Los (*n* = 12), and Ovx–Los (*n* = 12) animal groups; **a** distance traveled during the EPM test. **b** Number of entries into the open arms; Ovariectomy reduced the number of entries into the open arms in Veh-treated rats; *p < 0.05 versus Sham, same treatment (Student’s *t* test). **c** Amount of time spent in the open arms; Ovariectomy reduced the amount of time spent in the open arms in Veh-treated rats; *p < 0.05 versus Sham, same treatment (Student’s *t* test)

Five weeks after ovariectomy, anxiety-like behavior was assessed in the EPM, which is based on natural rodent behaviors including exploration and avoidance of open areas and height (Korte and De Boer 2003). Ovariectomy had no effect on the distance traveled in the EPM (Fig. 2a) indicating there were no impairments in spontaneous motor activity. Ovariectomy reduced the number of entries (*p* < 0.05) (Fig. 2b) and percentage of time spent (*p* < 0.05) (Fig. 2c) in the open arms. These anxiety-like behavioral effects of ovariectomy were prevented by Los treatment.

### Effect of Ovariectomy and Losartan on Open Field Test Behavior

**Fig. 3:**
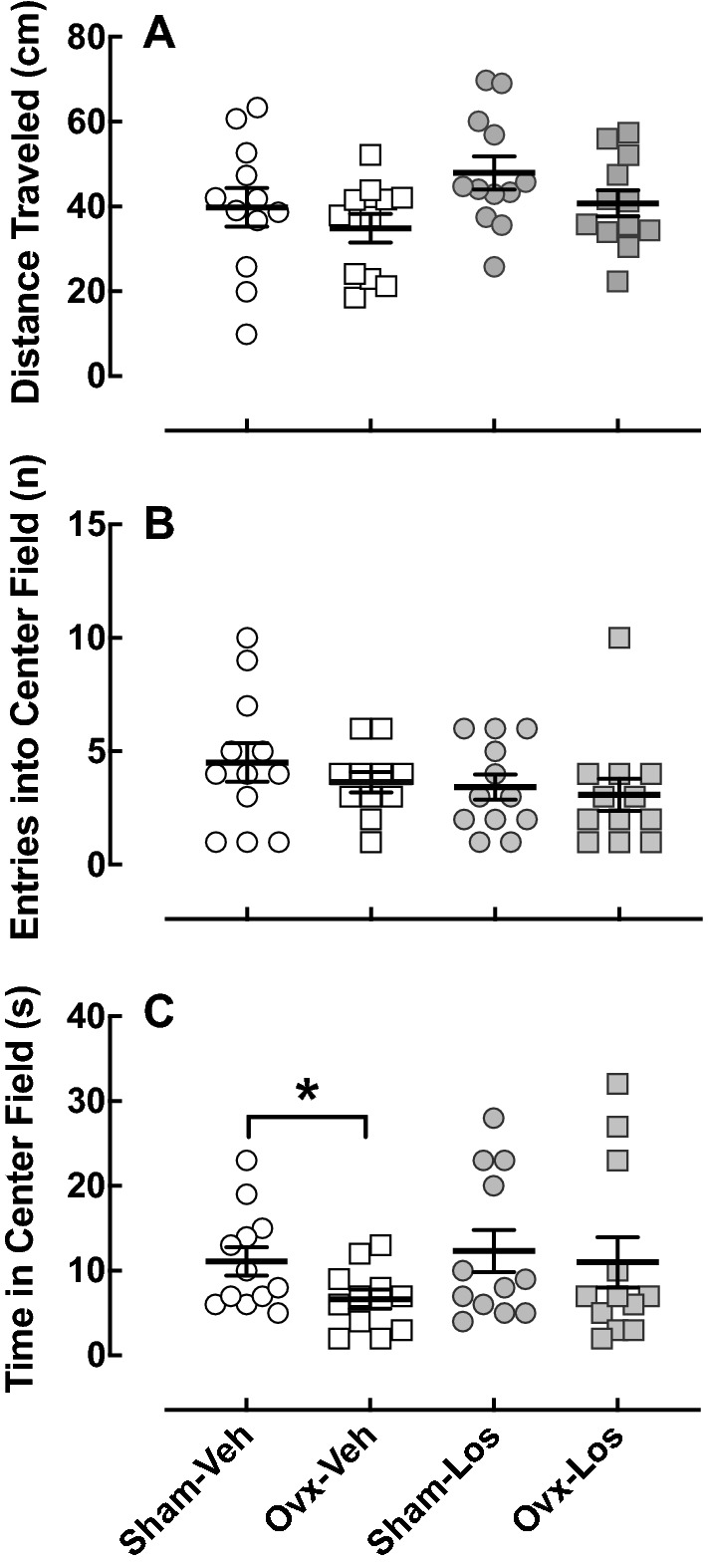
Effect of ovariectomy and losartan on behavior in the open field test. The OF test was assessed in Sham–Veh (*n* = 12), Ovx–Veh (*n* = 11), Sham–Los (*n* = 12), and Ovx–Los (*n* = 12) animal groups; **a** Distance traveled during the EPM test. **b** Number of entries into the center field. **c** Amount of time spent in the center field; Ovariectomy reduced the time spent in the center field in Veh-treated rats; *p < 0.05 vs. Sham, same treatment (Student’s *t* test)

Five and a half weeks after ovariectomy, anxiety was assessed in the OF test, which is based on natural rodent behaviors including exploration and avoidance of open areas (Korte and De Boer 2003). Ovariectomy had no effect on the distance traveled in the OF (Fig. 3a) indicating there were no impairments in spontaneous motor activity. Although there was no effect of ovariectomy on the number of entries into the center field in the Veh-treated group (Fig. 3b), ovariectomy did reduce the time spent in the center of the field (*p* < 0.05) (Fig. 3c). This anxiety-like behavioral effect of ovariectomy was prevented in the Los-treated animals.

### Effect of Ovariectomy and Losartan on the Novel Object Recognition Test

**Fig. 4:**
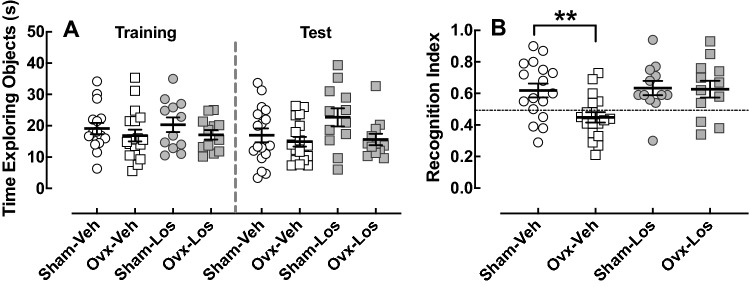
Effect of ovariectomy and losartan on novel object recognition. The NOR test was assessed in Sham–Veh (*n* = 17), Ovx–Veh (*n* = 18), Sham–Los (*n* = 12), and Ovx–Los (*n* = 12) animal groups. **a** Amount of time spent exploring objects during the training and test periods. **b** Recognition index for the amount of time spent with the familiar versus novel object. Ovariectomy reduced the recognition index in Veh-treated rats; **p < 0.01 versus Sham, same treatment (Student’s *t* test)

Six weeks after ovariectomy, cognition was assessed by the NOR test, which is based on the natural behavior of rodents to explore new objects compared to familiar ones and is widely used to assess cognitive impairments in rodent models of disease (Grayson et al. 2015). The longer time spent investigating the novel object in comparison with the familiar object indicates short-term memory recognition. There was no effect of ovariectomy on the percentage of total time spent exploring the objects during the training and testing phase (*p* < 0.05) (Fig. 4a) indicating there were no impairments in spontaneous motor activity or interest in exploring objects. However, ovariectomy reduced the recognition index in the Veh-treated animals to values below 0.5 (*p* < 0.05) (Fig. 4b), suggesting they did not recognize the familiar object. This impaired cognitive effect of ovariectomy was prevented in the Los-treated animals.

### Effect of Ovariectomy and Losartan on the Looming Visual Stimuli Test

**Fig. 5:**
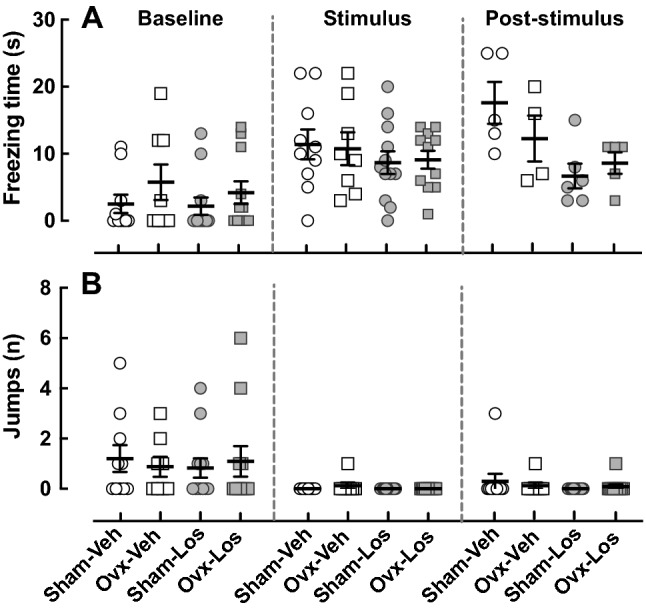
Effect of ovariectomy and losartan on behavior in the looming visual stimuli test. The LVS test was assessed in Sham–Veh (*n* = 10), Ovx–Veh (*n* = 8), Sham–Los (*n* = 12), and Ovx–Los (*n* = 11) animal groups. **a** Freezing behavior time during the pre-test, test, and post-test phases. Los treatment extinguished the freezing behavior more rapidly than the Veh-treated animals regardless of surgery in the poststimulus period; *p* < 0.02 by two-way ANOVA (surgery, treatment) *F* = 8.53; DFn = 1; DFd = 16. **b** Number of jumps during the pre-test, test, and post-test phases

Seven and a half weeks after ovariectomy, defensive responses were assessed in the LVS test, which is based on a rodent’s reflexive behavior in response to potential threat conditions and includes freezing (total absence of movement of the body, attentive attitude, head raised, arched back, retraction of the ears, eyes open, and piloerection) (Yilmaz and Meister 2013). No differences were observed between the Sham and Ovx animals in freezing behavior (Fig. 5a) or number of jumps (Fig. 5b) during the pre-test, test, and post-test periods. During the poststimulus phase, Los treatment extinguished the freezing behavior more rapidly than the Veh-treated animals (*p* < 0.02) (Fig. 5a).

### Effect of Ovariectomy and Losartan on Sucrose Preference

**Fig. 6:**
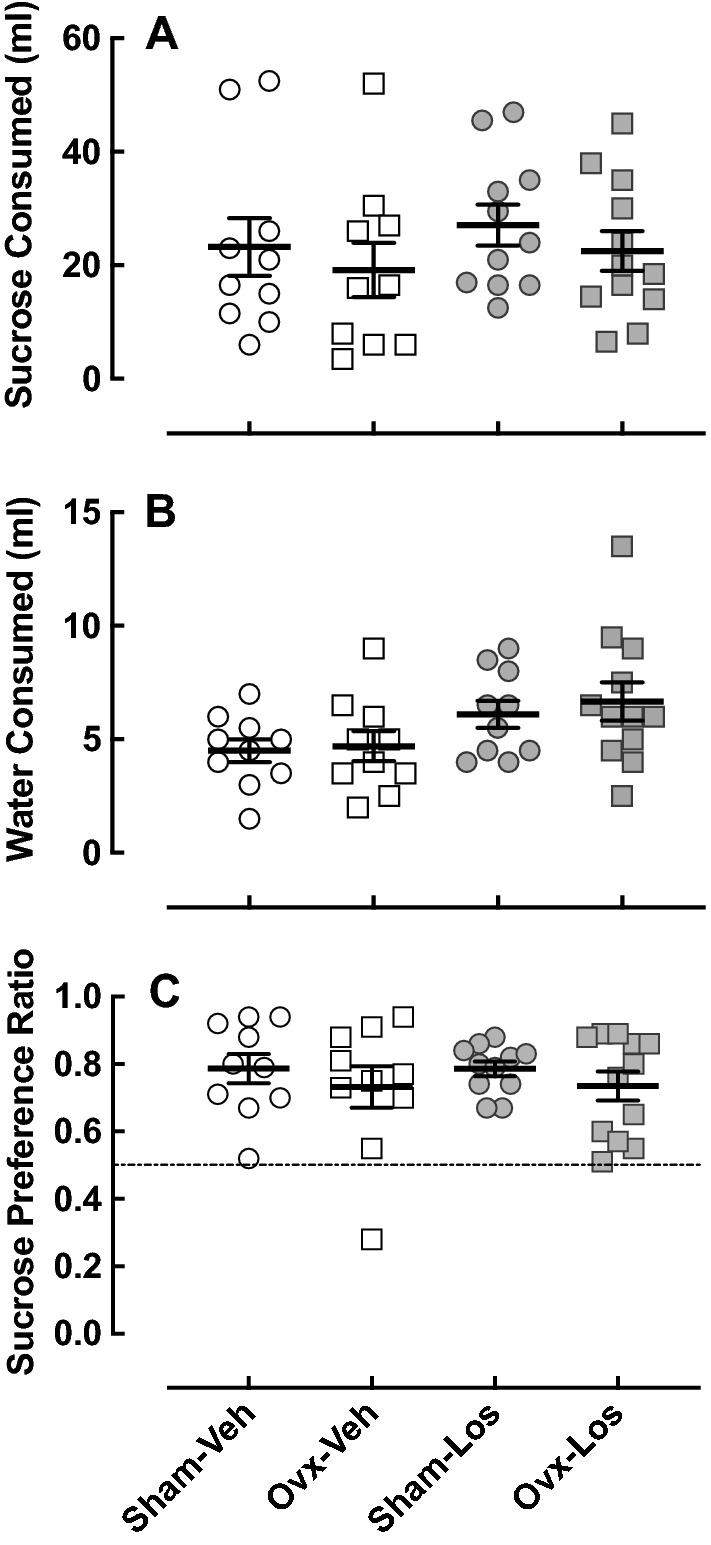
Effect of ovariectomy and losartan on sucrose preference. The sucrose preference test was assessed in Sham–Veh (*n* = 10), Ovx–Veh (*n* = 10), Sham–Los (*n* = 11), and Ovx–Los (*n* = 12) animal groups. **a** Daily amount of sugar water consumed. **b** Daily amount of tap water consumed. Los treatment caused an increase in tap water intake regardless of surgery; *p* < 0.02 by two-way ANOVA (surgery, treatment), *F* = 6.70; DFn = 1; DFd = 38. **c** Ratio of sugar water to tap water consumed

Eight and a half weeks after ovariectomy, depression-like behavior was assessed in the SP test, which is based on a healthy rodent’s preference for drinking sweet solutions when given the choice over plain water (Sobrian et al. 2003). The absence of this preference is characteristic of anhedonic-like behavior. There was no effect of ovariectomy in the Veh-treated animals on sugar water (Fig. 6a) or tap water (Fig. 6b) intake or sucrose preference (Fig. 6c). While Los had no effect on sugar water intake or sucrose preference, there was a slight increase in tap water intake (*p* < 0.02) (Fig. 6b).

### Effect of Losartan on Plasma Corticosterone

Corticosterone is the primary adrenal steroid associated with stress in rodents because they lack the enzyme that converts corticosterone to cortisol (Gallo-Payet and Battista 2014). Thus, corticosterone was determined in the plasma 10 weeks after ovariectomy. There was a trend for ovariectomy to increase plasma corticosterone (Table 1). Furthermore, while Los treatment had no effect on the Sham animals, this ARB markedly reduced plasma corticosterone in the Ovx animals (*p* < 0.05) (Table 1).

### Effect of Ovariectomy and Losartan on Angiotensin Type 1 Receptor Expression in the Amygdala and Hippocampus

**Fig. 7:**
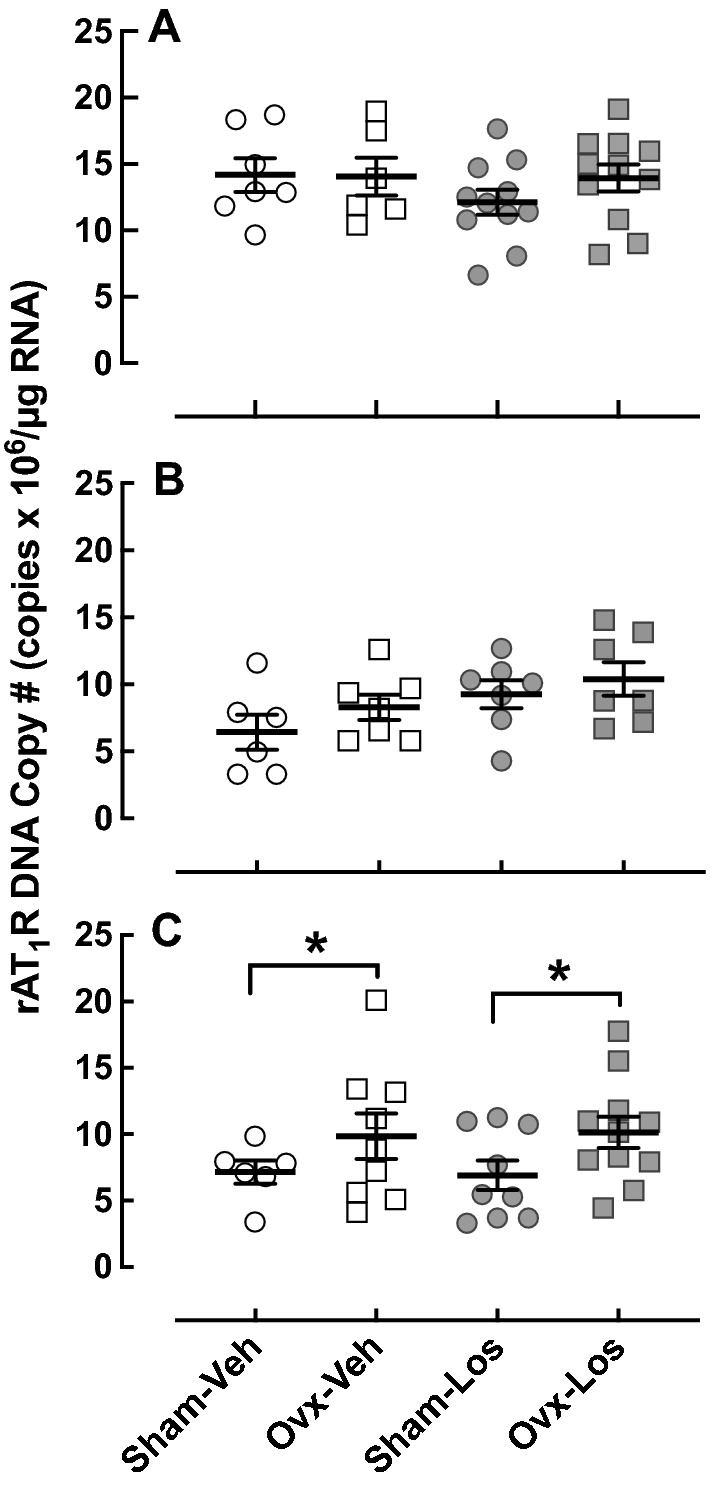
Effect of ovariectomy and losartan on AT_1_R mRNA expression in the BLA and HC. AT_1_R mRNA expression was determined by real-time PCR. **a** BLA [Sham–Veh (*n* = 7), Ovx–Veh (*n* = 6), Sham–Los (*n* = 11), and Ovx–Los (*n* = 11). **b** Hc CA1 [Sham–Veh (*n* = 6), Ovx–Veh (*n* = 7), Sham–Los (*n* = 7), and Ovx–Los (*n* = 7). Los increased AT_1_R mRNA regardless of surgery; *p* < 0.05 by two-way ANOVA (surgery, treatment) F = 4.74; DFn = 1; DFd = 23. **c** Hc CA3 [Sham–Veh (*n* = 6), Ovx–Veh (*n* = 9), Sham–Los (*n* = 9), and Ovx–Los (*n* = 11)]. Ovariectomy increased AT_1_R mRNA regardless of treatment; *p < 0.05 vs Sham, same treatment (Student’s *t* test); *p* < 0.05 by two-way ANOVA (surgery, treatment) F = 4.79; DFn = 1; DFd = 31

The amygdala contributes to anxiety-like behavior in the EPM and OF tests (Tye et al. 2011) while the hippocampus is involved in the NOR task (Cohen and Stackman 2015). Thus, we investigated the effects of ovariectomy on AT_1_R expression in these brain regions. There was no effect of ovariectomy on AT_1_R mRNA expression in the amygdala (Fig. 7a) or CA1 region of the hippocampus (Fig. 7b). In contrast, ovariectomy increased AT_1_R expression in the CA3 region (*p* < 0.05) (Fig. 7c). Losartan had no effect on AT_1_ mRNA expression in the amygdala or CA3 region of the Hc; however, Los increased AT_1_R mRNA in the CA1 region of the Hc (*p* < 0.05).

## Discussion

A major observation in this study was that Long Evans rats exhibited increased anxiety-like behavior in the EPM and OF tests after ovariectomy. Similar anxiety-like behaviors were shown in rats in the EPM (Zimmerberg and Farley 1993; Azizi-Malekabadi et al. 2015; Fedotova et al. 2017b), OF (Azizi-Malekabadi et al. 2015; Fedotova et al. 2017b) and light–dark exploration (Patki et al. 2013; Fedotova et al. 2017b) tests 4 weeks after ovariectomy. Furthermore, Schoenrock et al. (2016) investigated the behavior of 37 strains of inbred mice in the OF test and noted an overall increase in anxiety-like behavior 2 weeks after ovariectomy although they found the behaviors depended upon the genetic background.

A second major observation was that ovariectomized Long Evans rats were unable to discriminate the new from the previously viewed object in the NOR test. Similar cognitive dysfunction was observed in ovariectomized Sprague–Dawley (Gibbs and Johnson 2008; Wallace et al. 2006; Gogos et al. 2018; Qu et al. 2013; Kim et al. 2016) and Wistar (Patki et al. 2013) rats. Ovariectomy caused impairments in the 12-arm radial maze (Gibbs and Johnson 2008), NOR test (Wallace et al. 2006; Gogos et al. 2018), Morris water maze (Qu et al. 2013; Kim et al. 2016; Patki et al. 2013) and passive avoidance tests (Gogos et al. 2018). Ovariectomy also impaired the cognitive performance of mice in NOR (Fonseca et al. 2013; Bastos et al. 2015; Monthakantirat et al. 2014), Morris water maze (Blair et al. 2016; Monthakantirat et al. 2014), step-through passive avoidance (Cai et al. 2013), and Y maze (Cai et al. 2013; Monthakantirat et al. 2014) tests.

These impairments in anxiety-like behavior and cognitive function produced in different rodent species (rats and mice) and strains (e.g., Long Evans and Sprague–Dawley rats and C57/Bl6, C58/Jj, SJL/J mice) and under various experimental conditions including the age of ovariectomy (8 weeks–9 months), the duration of ovariectomy (1 week to 15 months) and the types of tests assessing anxiety-like disorders (OF, EPM, Y maze, dark–light paradigm) and cognitive performance (NOR, Morris water maze, 12-arm radial maze, passive avoidance) indicates neurocognitive dysfunction in rodents is a widely observed response to sudden ovarian hormone loss-induced by ovariectomy. Thus, the ovariectomized rodent is a useful model for investigating therapeutic strategies for preventing neurocognitive dysfunction that is associated with bilateral oophorectomy in women. Moreover, strain-dependent differences in the degree of dysfunction reflect the human population and can be exploited when designing studies investigating susceptibility and resilience to ovariectomy-induced neurocognitive disorders.

Ovariectomy did not show any effects in the LVS test. To our knowledge, this is the first time that ovariectomized rodents have been assessed in the LVS test. The lack of effect may be due to a ceiling effect since the intact animals stayed frozen during the entire test period and for most of the poststimuli period. Aguilar et al. (Aguilar et al. 2018) showed intact Sprague–Dawley rats under the same LVS protocol exhibited a freezing response profile that was similar to our findings in the Long Evans rats. We cannot rule out the possibility that other tests of fear conditioning might reveal an inhibitory effect of ovariectomy on fear extinction in these normotensive rats.

We did not find an effect of ovariectomy on the SP test. Both Sham and Ovx animals showed a clear preference for drinking sweet water over tap water. Our results are supported by previous reports in rodents showing ovariectomy had no effect on the SP test unless the rodents were subjected to forced swimming (Gogos et al. 2018; Bastos et al. 2015), tail suspension (Bastos et al. 2015), or chronic mild stress (Rygula et al. 2008; Romano-Torres and Fernandez-Guasti 2010; Huang et al. 2015). These findings suggest ovariectomy in the absence of a stressor is insufficient to cause anhedonic-like behavior in young adult rats. However, it is also possible that another behavioral test of anhedonic-like behavior would detect depressive-like symptoms induced by ovariectomy in these rats.

Not all women develop hypertension after bilateral oophorectomy. Similarly, while ovarian hormone loss is often associated with increased BP, the effect on BP depends upon the animal model (Sandberg and Ji 2012). Ovariectomy was shown to increase BP in the spontaneous hypertensive rat, Dahl salt-sensitive rat, and mRen2-Lewis rat, among others (Sandberg and Ji 2012). In contrast, ovariectomy did not increase BP in Dahl salt-resistant rats (Zheng et al. 2008; Pai et al. 2018). In this study, we found Long Evans rats also remained normotensive and their MAP was not elevated compared to sham-operated animals 6 weeks after ovariectomy. Therefore, the Long Evans rat is a valuable normotensive model in which to study anxiety-like behavior and cognitive dysfunction induced by ovarian hormone loss in the absence of BP modulation by ovariectomy.

The major finding of our study was that Los prevented anxiety-like behavior and cognitive impairments in a normotensive animal. These observations support previous studies indicating ARBs have BP-independent neuroprotective effects. Much literature points to a positive correlation between hypertension and neurocognitive disorders. Individuals with hypertension performed worse on tasks that measured speed of information processing, short-term memory, and reaction time and they reported higher levels of anxiety than their normotensive counterparts (Blumenthal et al. 1993). Furthermore, an early placebo-controlled study of systolic hypertension in Europe found there was a lower incidence of dementia in individuals on antihypertensive treatment compared to placebo controls (Forette et al. 1998). These clinical studies suggest the neuroprotective effects of ARBs are in part due to their antihypertensive effects. However, the magnitude of ARB neuroprotection varied independently of their effectiveness for lowering BP (Yasar et al. 2013; Davies et al. 2011; Johnson et al. 2012; Li et al. 2010). Thus, clinical findings suggest that ARBs exert neuroprotection via BP-dependent and BP-independent pathways. Studies in animals support these findings. Treatment with an ARB improved age-associated memory dysfunction in a passive avoidance test in male rats under conditions in which BP was not reduced (Hirawa et al. 1999). Similarly, ARBs attenuated hypertension-associated cognitive dysfunction in male rats at doses that did not alter BP (Pelisch et al. 2011). ARBs also exerted neuroprotective effects on cognition independently of BP alterations in a male model of postoperative cognitive dysfunction (Li et al. 2014).

Bilateral oophorectomy is strongly associated with BW gain in humans and animals (Roepke 2009) and is a widely reported effect of E_2_ deficiency (Gonzalez-Garcia et al. 2017). Accordingly, we found Ovx rats gained more than twice as much weight compared to Sham animals over the 10-week experiment. Excessive BW is a risk factor for mood disorders (Rocca et al. 2009) and cognitive impairment (Zanini et al. 2017) in humans and animals (Zanini et al. 2017). However, it has been difficult to tease apart BW gain from ovarian hormone loss in the mechanisms contributing to Ovx-induced neurocognitive dysfunction. Ovx-induced BW gain can be prevented with E_2_ replacement (Hinojosa-Laborde et al. 2004; Roesch 2006) and E_2_ treatment is cognitively protective (Fonseca et al. 2013). Exercise, which can reduce body weight and alter body composition, also has neurocognitive protective effects (Kim et al. 2016). Therefore, our finding that Los prevents anxiety-like behavior and impairment of novel object recognition without reducing the Ovx-induced BW gain indicates the neuroprotective effects of this AT_1_R antagonist are either independent of neurocognitive dysfunction induced by excessive BW gain or the neuroprotective mechanisms of Los are downstream from those causing BW gain.

Our finding that plasma corticosterone levels were lower in Ovx rats treated with Los compared to Veh-treated Ovx animals and that Los also attenuated the freezing behavior during the poststimulus period of the LVS test suggests blocking AT_1_Rs reduces stress pathways and facilitates fear extinction. These observations support previous studies in male rats showing that blockade of central AT_1_Rs with the ARB candesartan decreased corticotrophin releasing factor (CRF) responses to stress (Armando et al. 2001, 2007). These studies showed candesartan reduced the expression of CRF and circulating corticosterone.

One explanation for our finding that Los treatment reduced plasma corticosterone in Ovx but not in the Sham animals is that Los selectively reduced hypothalamic CRF production induced by AT_1_Rs activated by ovariectomy. Activation of AT_1_Rs is known to induce the synthesis and secretion of hypothalamic CRF leading to corticosterone release (Raasch et al. 2006). Thus, increased activity of Hc CA3 AT_1_Rs contributes to stress-induced stimulation of the hypothalamic pituitary axis. In addition, studies have shown increased levels of corticosterone is associated with impaired learning and memory in rodents and humans (Joels et al. 2018). Therefore, inhibition of the corticosterone stress response may also contribute to the ability of Los to prevent cognitive deficits in the NOR test.

The observation that ovariectomy increased AT_1_R expression in the CA3 region of the Hc extends previous reports showing ovariectomy upregulates AT_1_R expression in other angiotensin II target tissues (Hinojosa-Laborde et al. 2004; Owonikoko et al. 2004). Activation of AT_1_Rs is known to increase oxidative stress, which in turn can induce cell death (Jackson et al. 2018). Ovariectomy was shown to increase markers of oxidative stress and apoptotic injury in primary rat hippocampal neurons (Yazgan and Naziroglu 2017) and to increase the number of apoptotic cells in CA3 (Sales et al. 2010; Peng et al. 2012). The CA3 region of the Hc is known to be involved in memory and cognition (Rolls 2013). Thus, the ovariectomy-induced increase in AT_1_R expression in the CA3 region of the Hc could contribute to cognitive impairment through increased oxidative stress and cell death. Taken together, these observations suggest the ability of this ARB to protect novel object recognition in the NOR test involves blockade of increased Hc CA3 AT_1_R activity.

Every year approximately 600,000 women elect bilateral oophorectomy in the USA (Rocca et al. 2009). These women are most often younger than the natural age of menopause (Armstrong et al. 2004). Our findings that ARBs prevent both anxiety-like behavior and cognitive decline induced by ovariectomy have immediate translational value for women who have bilateral oophorectomies. Thus, it will be worthwhile to investigate the neuroprotective effects of ARBs in controlled clinical trials in women who undergo surgical menopause prior to the natural age of menopause and to delve into the mechanisms underlying Los neurocognitive protection.

The neuroprotective effects of ARBs may also extrapolate to other populations of women who are ovarian hormone deficient. Premature ovarian failure is a syndrome defined as a non-physiological cessation of ovarian function before 40 years of age in contrast to 51, the average age of menopause (de Moraes-Ruehsen and Jones 1967). The hormonal profile of these women is similar to what occurs during the early physiological postmenopausal phase and is characterized by low levels of estrogen and high levels of follicle-stimulating hormone (Jankowska 2017; Barrett et al. 2019). Although women with premature ovarian failure do not have the sudden drop in ovarian hormones that women who undergo bilateral oophorectomies experience, studies in rats and mice suggest symptoms of anxiety are worse the longer the duration of ovarian hormone deficiency (Lagunas et al. 2010; de Chaves et al. 2009; Citraro et al. 2015). Daendee et al. (Daendee et al. 2013) conducted a time course of the effects of ovariectomy duration and showed that the percentage of rats exhibiting anxiety-like behavior in the elevated T maze increased from ~ 30 to ~ 60% and 90%, 2, 3, and 4 weeks after ovariectomy, respectively. Thus, ARBs may also be neuroprotective in women with premature ovarian failure given that their life-long exposure to ovarian hormone deficiency is increased compared to women who experience normal menopause.

While many drugs are used to treat anxiety including benzodiazepines, selective serotonin reuptake inhibitors, serotonin norepinephrine reuptake inhibitors, tricyclic antidepressants, or monoamine oxidase inhibitors (Fedotova et al. 2017a), they are often associated with adverse side effects that negatively impact one’s basic activities and general quality of life (Koen and Stein 2011; Trindade et al. 1998). Unfortunately, there are no particularly efficacious drugs on the market for preventing cognitive decline in individuals with mild cognitive impairment or AD (Atri 2019). Thus, a great advantage of using ARBs as a new therapeutic strategy for neuropsychiatric disorders of anxiety and cognitive impairment is the low incidence of side effects, which makes these drugs generally well tolerated; adverse effects of ARBs occur in less than 10% of patients (Barreras and Gurk-Turner 2003).

In conclusion, our findings showed that the ARB Los prevented anxiety-like behavior and memory impairments induced by ovariectomy in Long Evans rats. These neuroprotective effects of Los occurred under normotensive conditions and were independent of ovariectomy-induced BW gain. The ability of Los to lower circulating levels of corticosterone in the Ovx rat suggests the neurocognitive protective effects of this ARB are in part due to reducing activation of stress pathways. Furthermore, the ovariectomy-induced increases in AT_1_R expression in the CA3 region of the Hc suggests blocking the activity of increased AT_1_Rs in this region contributes to the neuroprotective effects of Los. This study can inform the design of controlled clinical trials aimed at testing the neurocognitive protective effects of ARB therapy in normotensive women who are at higher risk of developing neuropsychiatric dysfunction because they had bilateral oophorectomies prior to natural age of menopause.
